# 

**Published:** 1901-07

**Authors:** 


### MARRIAGES.

ON May 20th, at Owosso, Michigan, Dr. William Jopling, an active member of the Michigan State Veterinary Medical Association, was married to Mrs. Emma A. Robbins, of the same city.

### ASSOCIATION MEETINGS.

The Veterinary Medical Association of New Jersey will hold their semi-annual meeting on Thursday, July 11th, at Stettler's Assembly Hall, 844 Broad Street (adjoining Broad Street Station), Newark, N. J. A surgical clinic is to be made a feature of this meeting, and the President's determination that "less time be spent in the transaction of routine business and more time devoted to the consideration of scientific and practical problems of the profession" will be carried out as far as possible.

GEORGE W. POPE,
Secretary.

### PERSONALS.

Dr. J. Cheston Morris, of Philadelphia, never loses his interest in veterinary science, and is always a welcome visitor to veterinary meetings in Philadelphia. His discussion and knowledge of comparative medicine are always interesting and entertaining.

Drs. Eves, Mahaffy, and Cole filled the rôle of veterinarians to the Wilmington, Delaware, horseshow, in June.

Dr. William Gall, of Matawan, N. J., met with a serious accident by fire, losing all his instruments, books, and much more of value.

Dr. Oscar Seeley, of Philadelphia, will be among the exhibitors at the Maryland Horseshow in the several saddle classes.

Veterinarians William Mole and F. H. Gallanough, of Toronto, Canada, fill the rôle of veterinary surgeons to the City Dairy Company of Spadina Crescent, which is furnishing the above city with milk prepared under special sanitary precautions.

Drs. Leonard Pearson, of Philadelphia, and P. K. Jones, of Wellsburg, Va., were in attendance at the Coopersburg sale of Channel Island cattle on Decoration Day. Dr. Jones purchased a number of very choice Jerseys for their new stock-farm enterprise.

Dr. F. H. Mackie, of Baltimore, was the official veterinarian to the Baltimore Horseshow in May.

Dr. Leonard Pearson, of Philadelphia, was a visitor to Montrose, Pa., early in May.

Dr. J. R. Mehaffy, of Wilmington, Delaware, entertained several of his colleagues of Philadelphia in May after a visitation to Temple Lodge, F. and A. M., of which Dr. Mehaffy is a floor officer.

Dr. Charles A. Dohan, one of the veterinarians to the Philadelphia Horseshow, was taken ill during the show and was absent from his post.

Dr. Charles T. Goentner, of Bryn Mawr, Pa., is much improved in health.

Dr. Benjamin McInnes, who has been at Charleston, S. C., for twenty-six years, and who until recently was the only qualified veterinarian in South Carolina, was a visitor at the new JOURNAL

office the middle of June. Dr. McInnes was in Philadelphia on legal affairs, and was accompanied by his attorney, the Hon. George H. Moffatt, of the South Carolina Legislature.

Dr. M. J. Chrisman, of Sugar Grove, Pa., spent some three weeks in May on an inspection trip.

Dr. F. E. Anderson, of Findlay, Ohio, deals in fancy driving horses in connection with his practice.

Dr. Thomas B. Rayner, of Chestnut Hill, Philadelphia, Pa., is a very successful rose grower, and the grounds about his home had more than one hundred bushes in bloom in June.

Dr. G. B. Brake, of Everett, Pa., is the only graduate of veterinary medicine in Bedford County. Dr. Brake was one of the class of 1890 at the Ontario Veterinary College.

Drs. Leonard Pearson and M. E. Conard will discuss several of the topics listed for consideration at the spring meeting of the Pennsylvania Board of Agriculture at Bellefonte, Pa., June 5th to 7th, inclusive.

Dr. James Cattanach, of New York, is about to go abroad, and will sell his famous trio of mules, with which he says he has driven many times more than sixty miles a day.

Dr. E. L. Cornman will locate in York, Pa., after a year's service as resident surgeon to the Veterinary Department of the University of Pennsylvania.

Dr. William H. Martinet, of Baltimore, Md., was a sojourner at Atlantic City the latter days of June, seeking a rest after the spring's arduous labors.

Dr. Leonard Pearson, State Veterinarian of Pennsylvania, may occasionally be seen on Philadelphia's streets with four very handsome and high-bred bull-terrier pups as companions in his peregrinations.

Dr. H. T. Carpenter, formerly practising at Ada, Ohio, has located at Lima, Ohio.

Dr. Oscar Seeley, of Philadelphia, was an exhibitor in the saddle classes at the Horseshow at Wilmington, Delaware, in June.

Dr. Pete McNeal, of Milton, Pa., spent several weeks in Philadelphia during the latter part of May and early part of June, taking a preparatory course for the State Board examination.

Dr. R. L. Kann, of Lisburn, Pa., a graduate of Ontario, after a third year at the McKillip Veterinary College, was under the special tutorship of Dr. J. M. Carter, of Philadelphia, preparatory to the State Veterinary Board examination.

Dr. Leonard Pearson, State Veterinarian, made a trip through Bradford County, and was in attendance at Bellefonte at the State Agricultural Board's session early in June.

Dr. Harry Walters and wife, of Trenton, N. J., spent the latter part of June at the Hotel Rudolf, Atlantic City.

Dr. J. B. Seitter, of Frankford, Philadelphia, Pa., has been filling the post of veterinarian during the illness of City Veterinarian Hart.

Dr. J. G. Ferneyhough, graduate of the United States College of Veterinary Surgeons, has removed from Somerset to Charlotteville, Va.

Dr. J. T. Ferley, Jr., of Philadelphia, takes an active part in several of the fraternal bodies of Pennsylvania, and has filled many of the higher offices in these organizations.

Dr. L. Sherman Cleaves, a graduate of the Veterinary Department of McGill University, has established the "Bar Harbor Veterinary Hospital," at Bar Harbor, Maine.

Dr. B. Howes is now located at Herr's Island, Allegheny, Pa., as an inspector of the Bureau of Animal Industry.

Veterinarian A. G. Hopkins, of Winnipeg, Manitoba, is now associate editor of the *Farmer's Advocate and Home Magazine*, an agricultural paper with a very large circulation throughout Canada.

Dr. W. Horace Hoskins, of Philadelphia, was a visitor to Harrisburg in the latter part of May in the interest of an appropriation for laboratory work under the Live-stock Sanitary Board.

Dr. H. Wellner, veterinarian of the Board of Health, Borough of Queens, New York City, was a recent caller at the new JOURNAL office. He stated that the newspaper reports of the vast epidemic among horses in New York was purely sensational, and was an advertising scheme, one of the prominent (?) veterinarians quoted being an illegal practitioner.

Dr. Louis Olry Lusson, of Ardmore, has just had a successful operation for cataract performed on both eyes. He has been obliged to withdraw from practice for some time on account of the trouble.

